# Supplementary material for: Dynamic genome evolution and complex virocell metabolism of globally-distributed giant viruses
Source: Nat Commun. 2020 Apr 6;11:1710. doi: 10.1038/s41467-020-15507-2 (PMC7136201; doi:10.1038/s41467-020-15507-2)
Supplement: Supplementary file 1 — Supplementary Information [file 41467_2020_15507_MOESM1_ESM.pdf]

Supplementary datasets and figures for Moniruzzaman et al., 2020.

Supplementary dataset descriptions

Supplementary Dataset 1. This spreadsheet contains summary information for each NCLDV MAG reported in this study, including the genome size, number of encoded proteins, metagenome of origin, and other data. Additionally, this spreadsheet contains contig-level summary statistics for all decontamination analyses used in this study, and a list of all contigs that were removed due to similarity to cellular or bacteriophage sources. Lastly, this sheet contains a list of all reference NCLDV genomes used in this study.

Supplementary Dataset 2. This spreadsheet contains the annotation information for the orthologous groups calculated in this study. Only orthologous groups that exhibited similarity to a known protein family are listed here. Also provided are the enriched COG categories identified in each NCLDV clade.

These datasets are available on the Aylward Lab Figshare account:

https://figshare.com/authors/Frank_Aylward/5008391 in the project titled “NCLDV”.

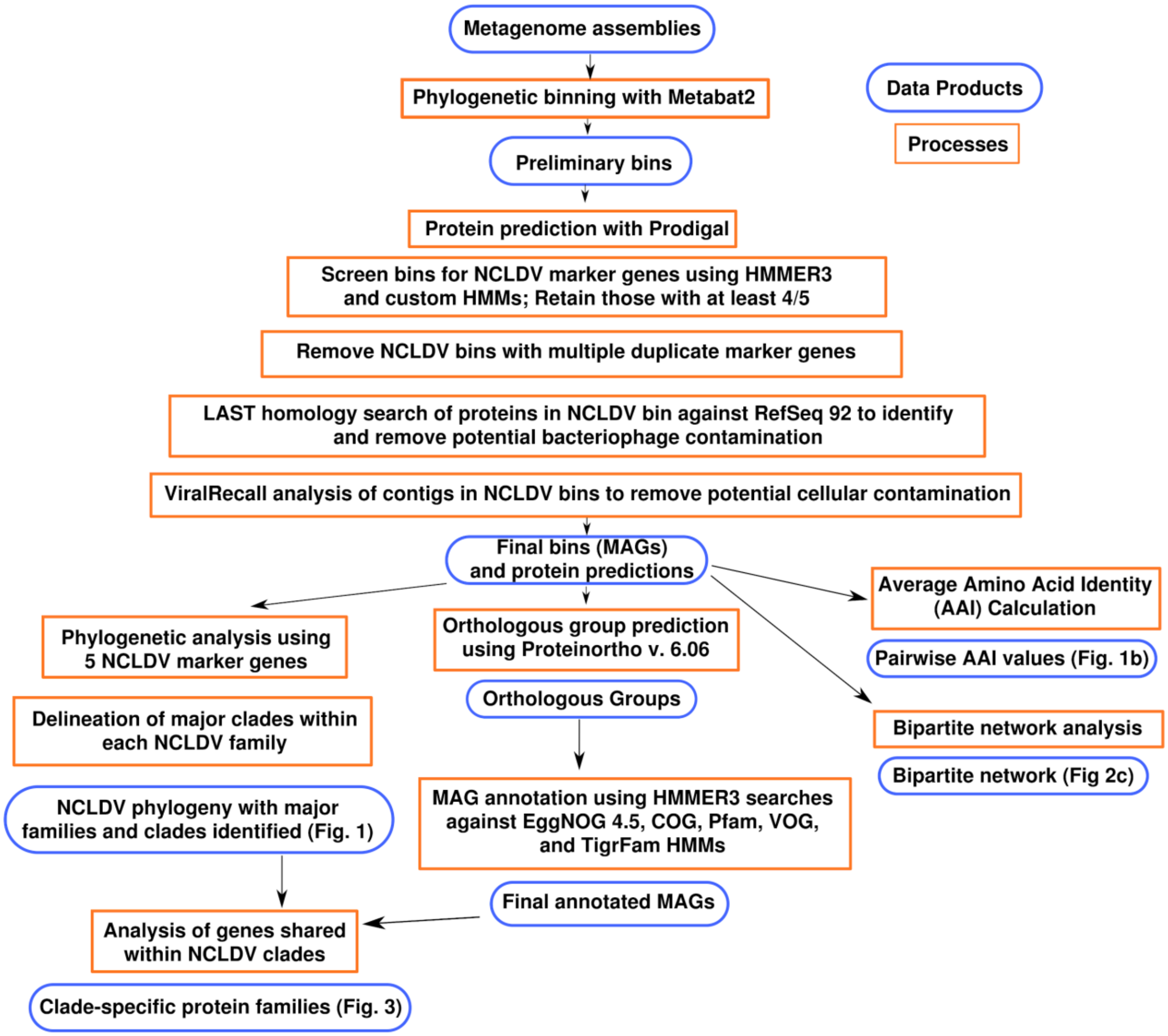

Supplementary Figure 1. Overview of the workflow used to identify and analyze the NCLDV MAGs described in this study.

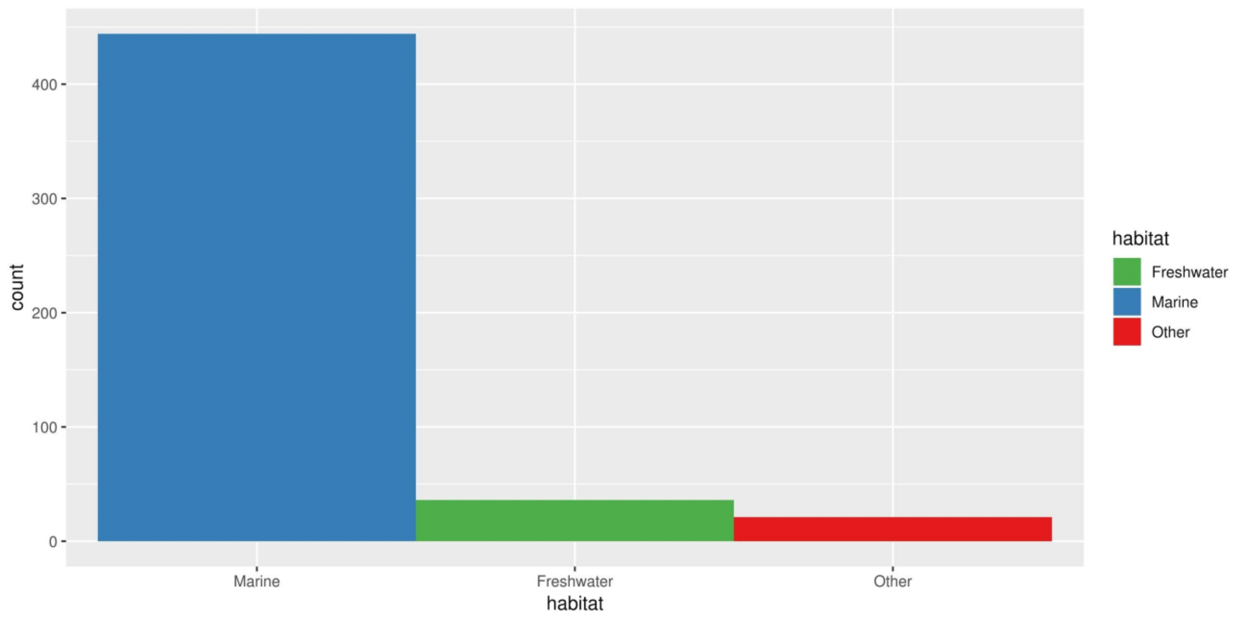

Supplementary Figure 2. Distribution of the habitats represented by the 501 NCLDV MAGs presented in this study.

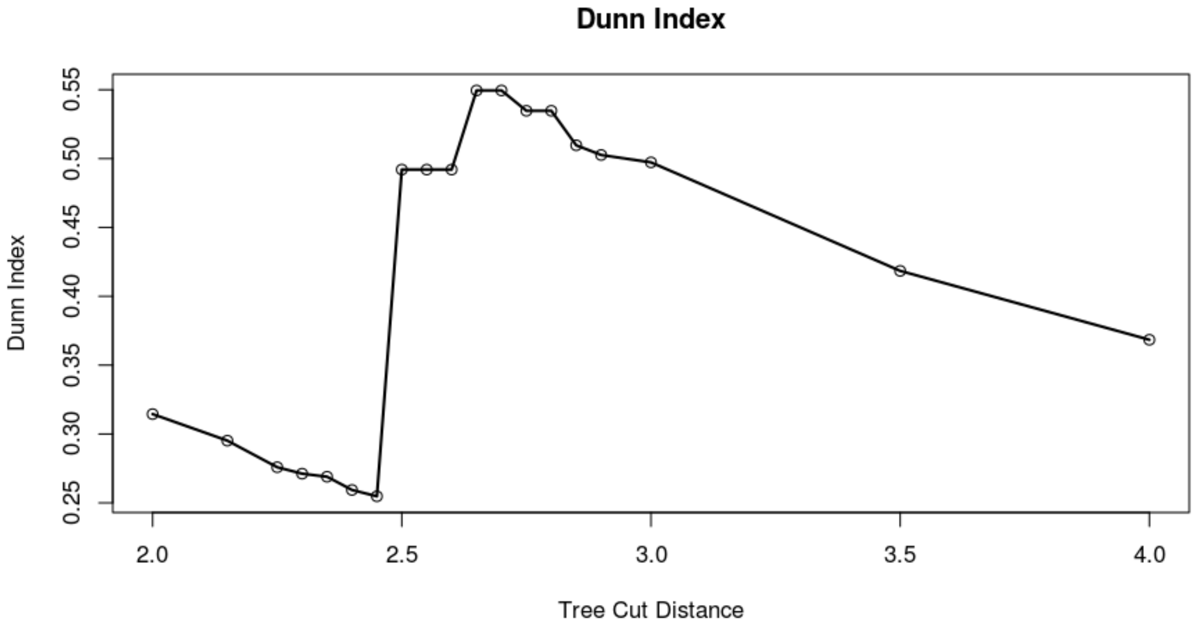

Supplementary Figure 3. Dunn index values for the different tree cut heights in our multi-locus NCLDV phylogeny. A cut height of 2.45 provided the best quality clade-level delineations, and these clusters were subsequently manually edited and inspected to provide the final NCLDV clades.

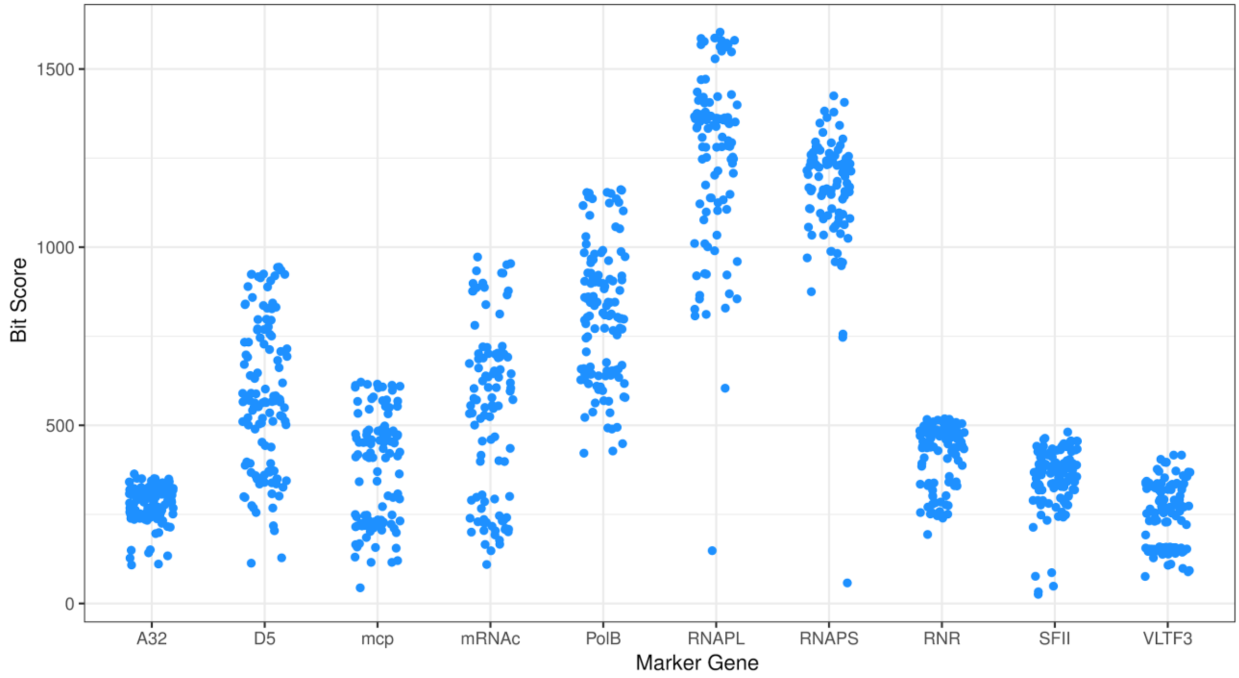

Supplementary Figure 4. Benchmarking of the Megavirales marker gene HMMs designed in this study. The bit scores are for the best hits recovered for each of 121 reference Megavirales genomes in NCBI GenBank. These HMMs were subsequently used to identify marker genes in the Megavirales MAGs generated in this study. The genes A32, MCP, PolB, SFII, and VLTF3 were subsequently used for phylogenetic analyses given they are broadly found across all Megavirales families.

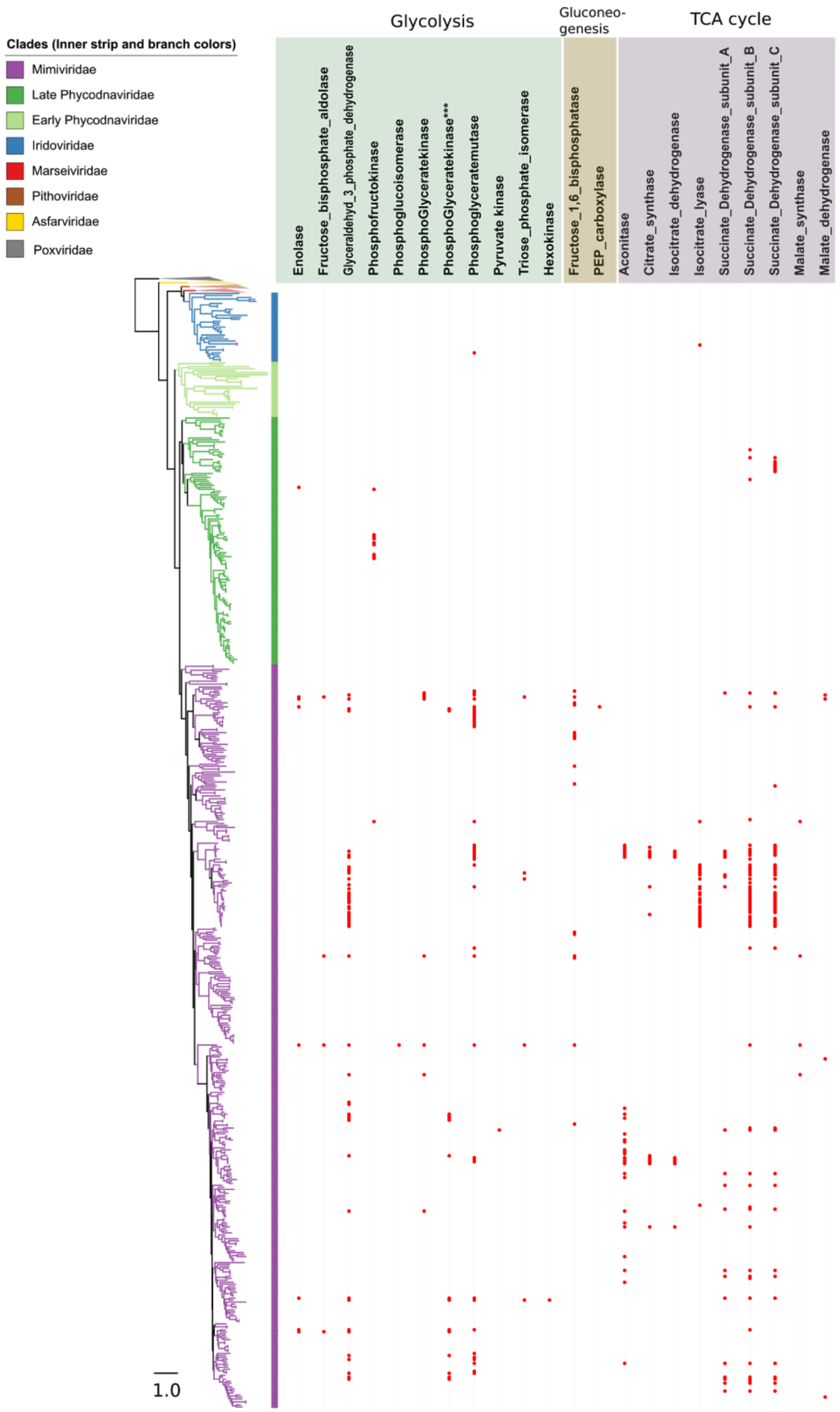

Supplementary Figure 5. Presence/absence of genes involved in Glycolysis, gluconeogenesis and TCA cycle in the NCLDV genomes. Pox-, Pitho- and Asfarviridae families were collapsed since none of these genes were found in the studied genomes from these families.

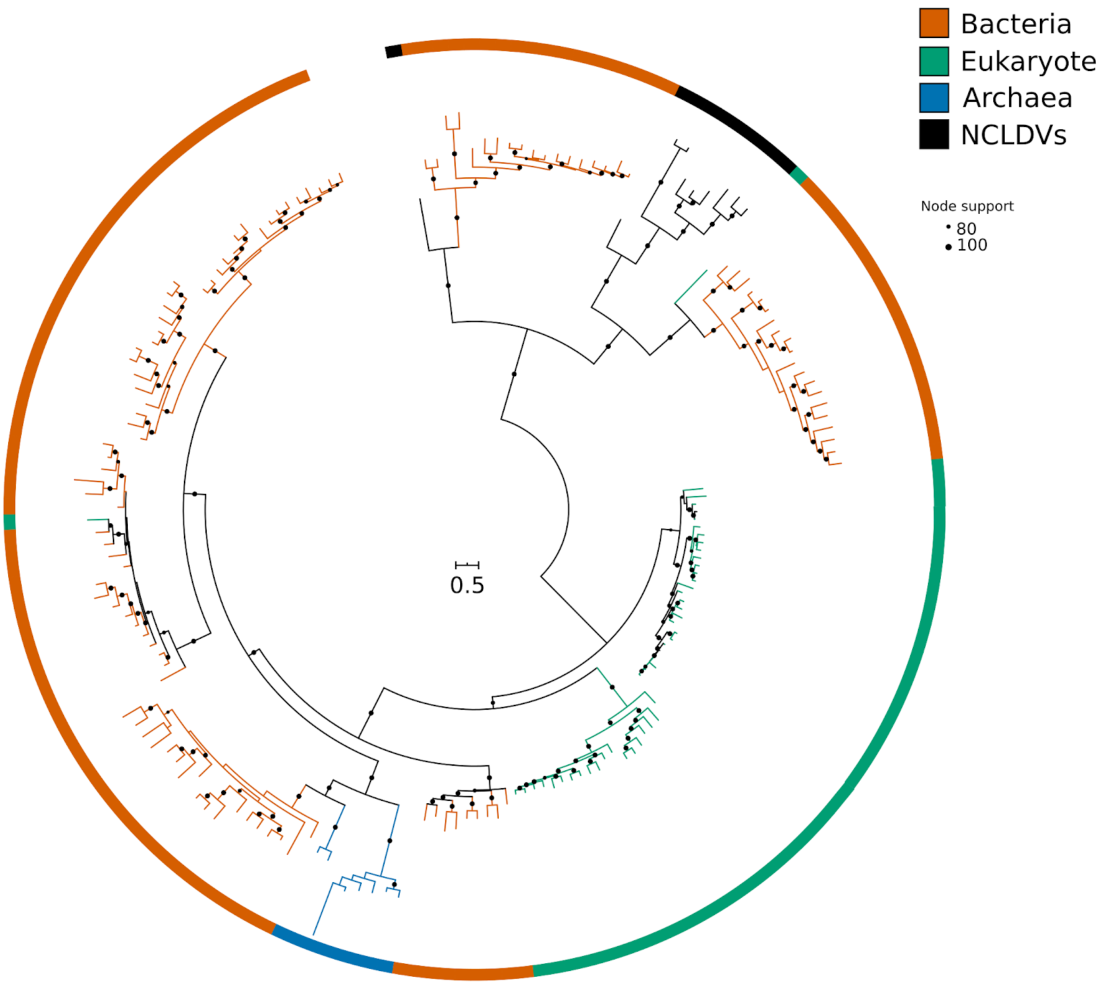

Supplementary Figure 6. Maximum-likelihood phylogenetic reconstruction of glycolytic enzyme phosphofructokinase. Ultrafast bootstrap support >80% are shown.

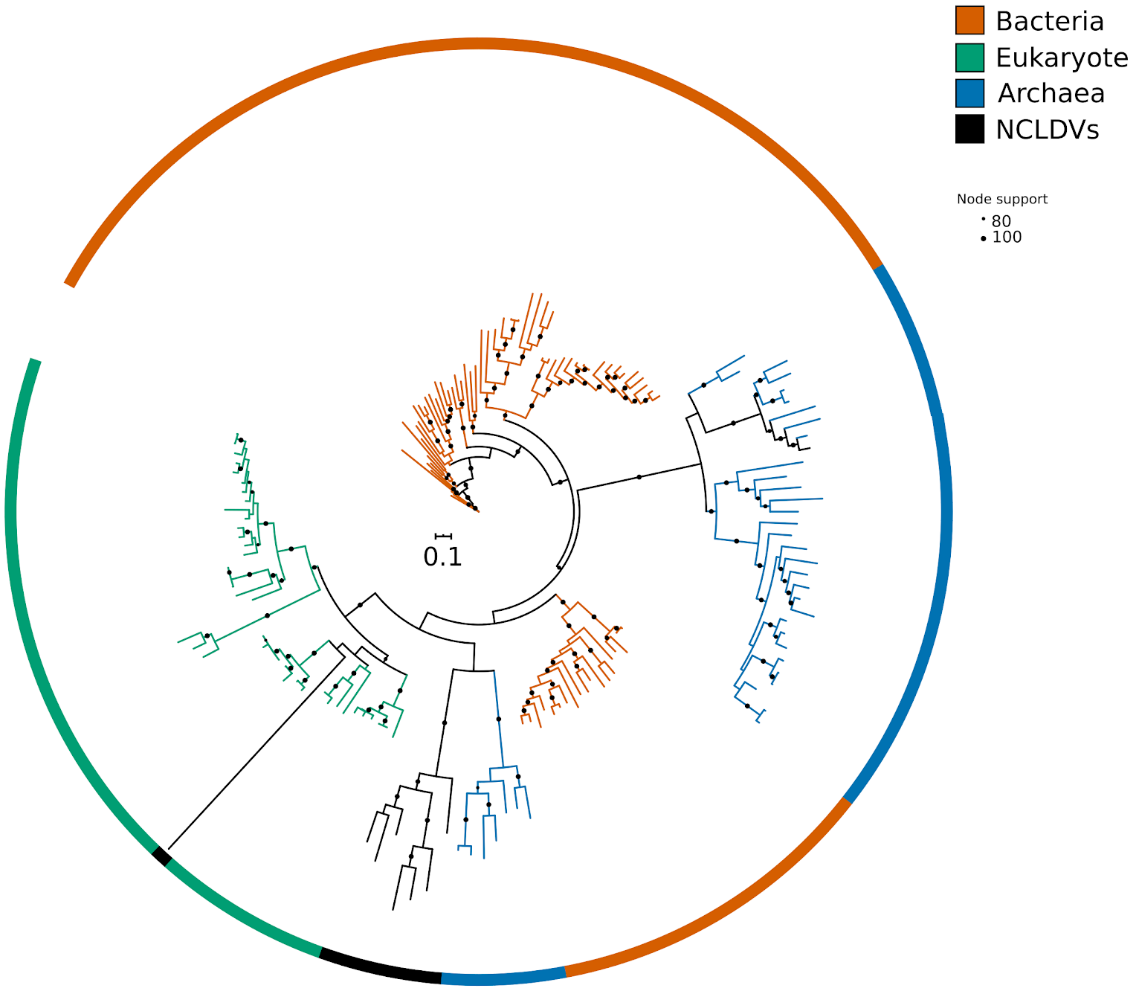

Supplementary Figure 7. Maximum-likelihood phylogenetic reconstruction of the glycolytic enzyme phosphopyruvate hydratase (enolase). Ultrafast bootstrap support >80% are shown.

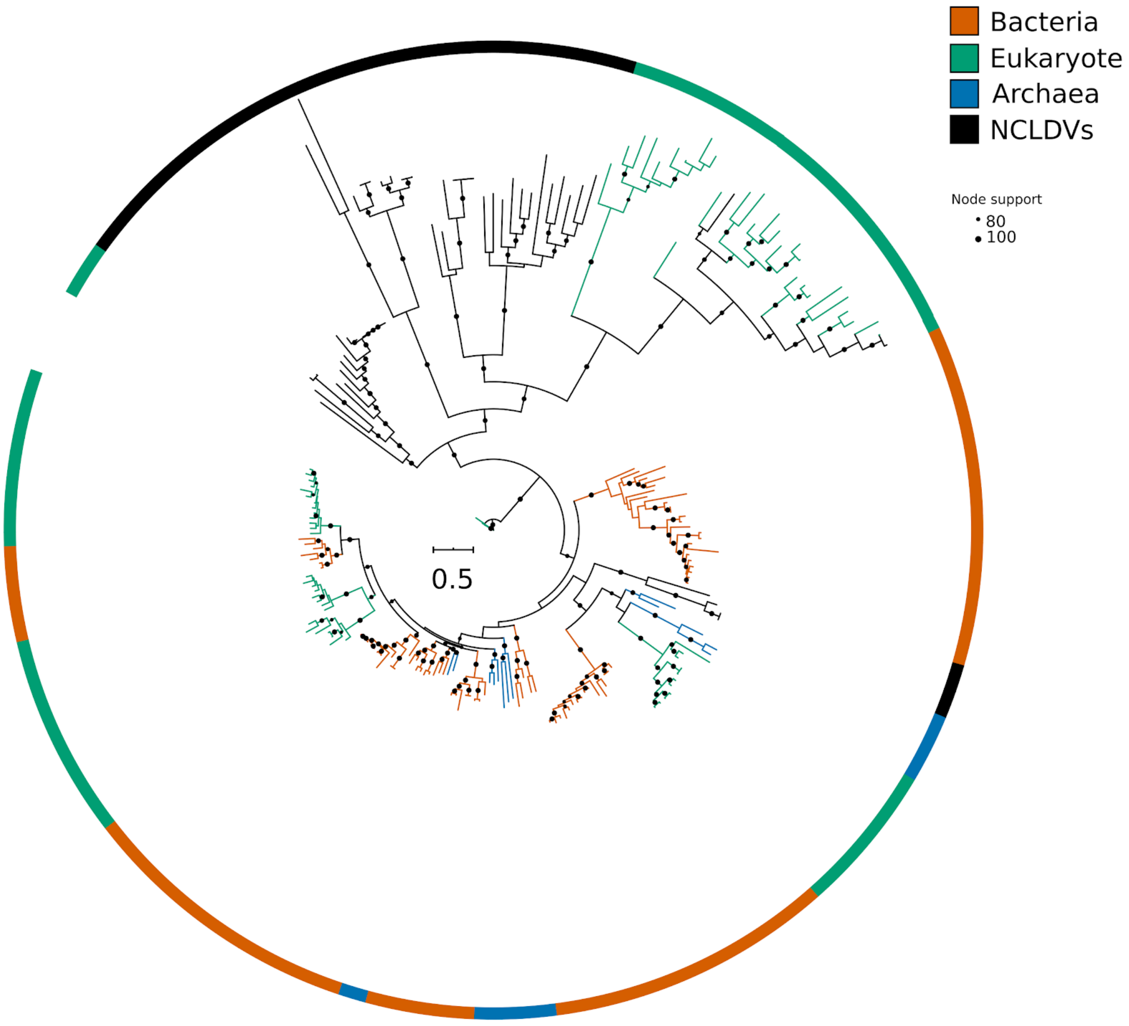

Supplementary Figure 8. Maximum-likelihood phylogenetic reconstruction of the glycolytic enzyme phosphoglycerate mutase. Ultrafast bootstrap support >80% are shown.

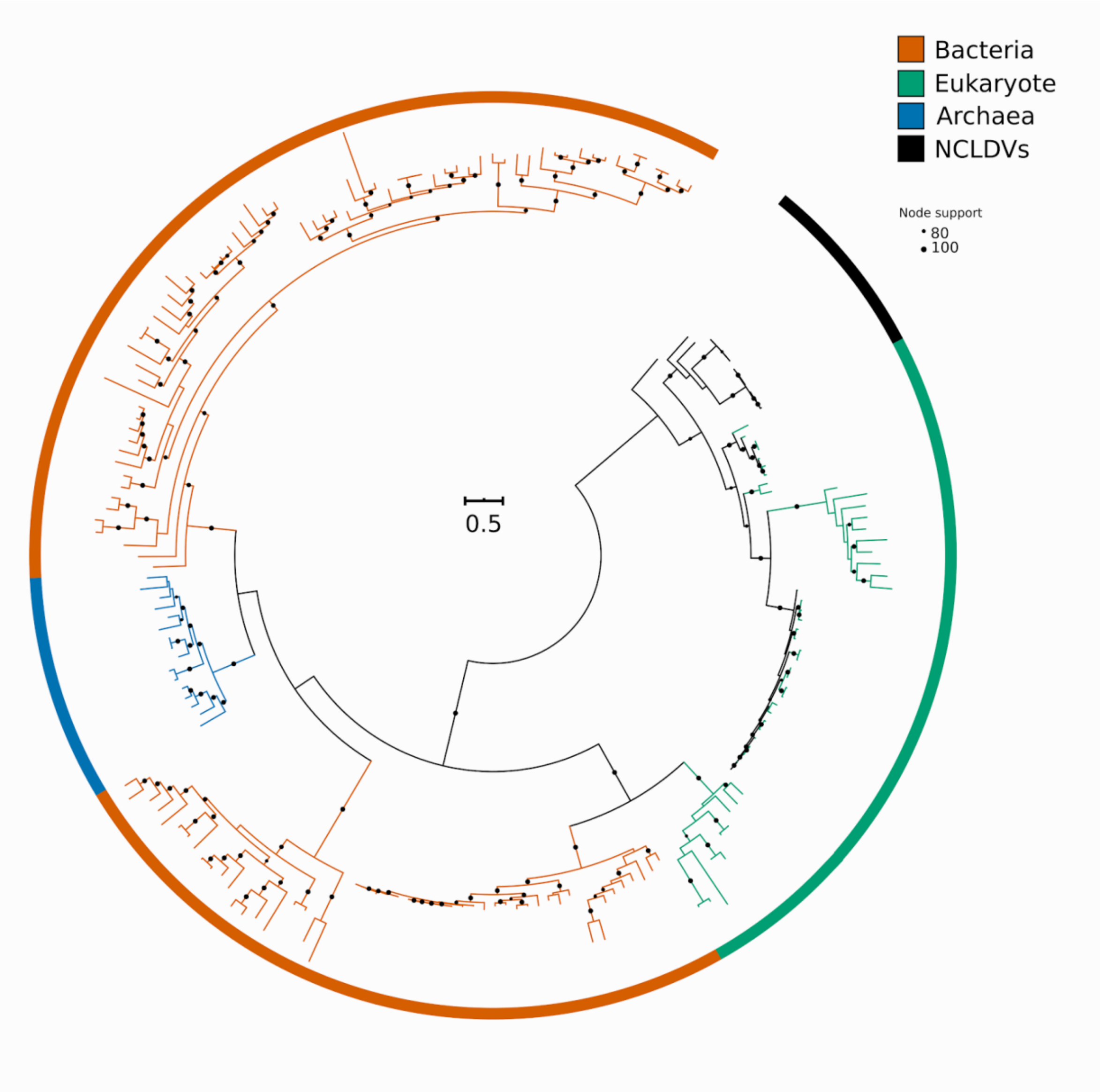

Supplementary Figure 9. Maximum-likelihood phylogenetic reconstruction of the TCA cycle enzyme citrate synthase. Ultrafast bootstrap support >80% are shown.

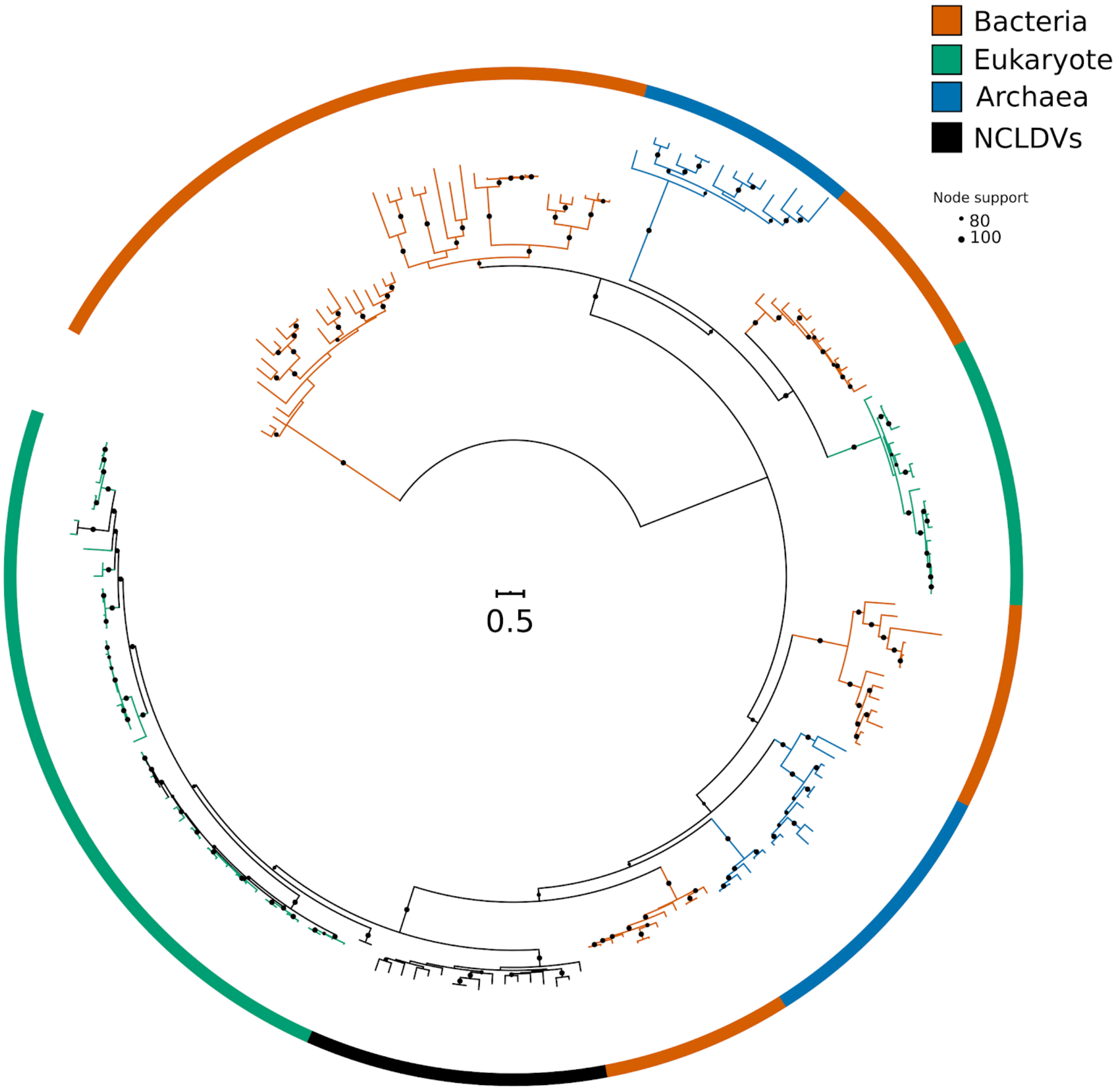

Supplementary Figure 10. Maximum-likelihood phylogenetic reconstruction of the TCA cycle enzyme Succinate dehydrogenase subunit A. Ultrafast bootstrap support >80% are shown.

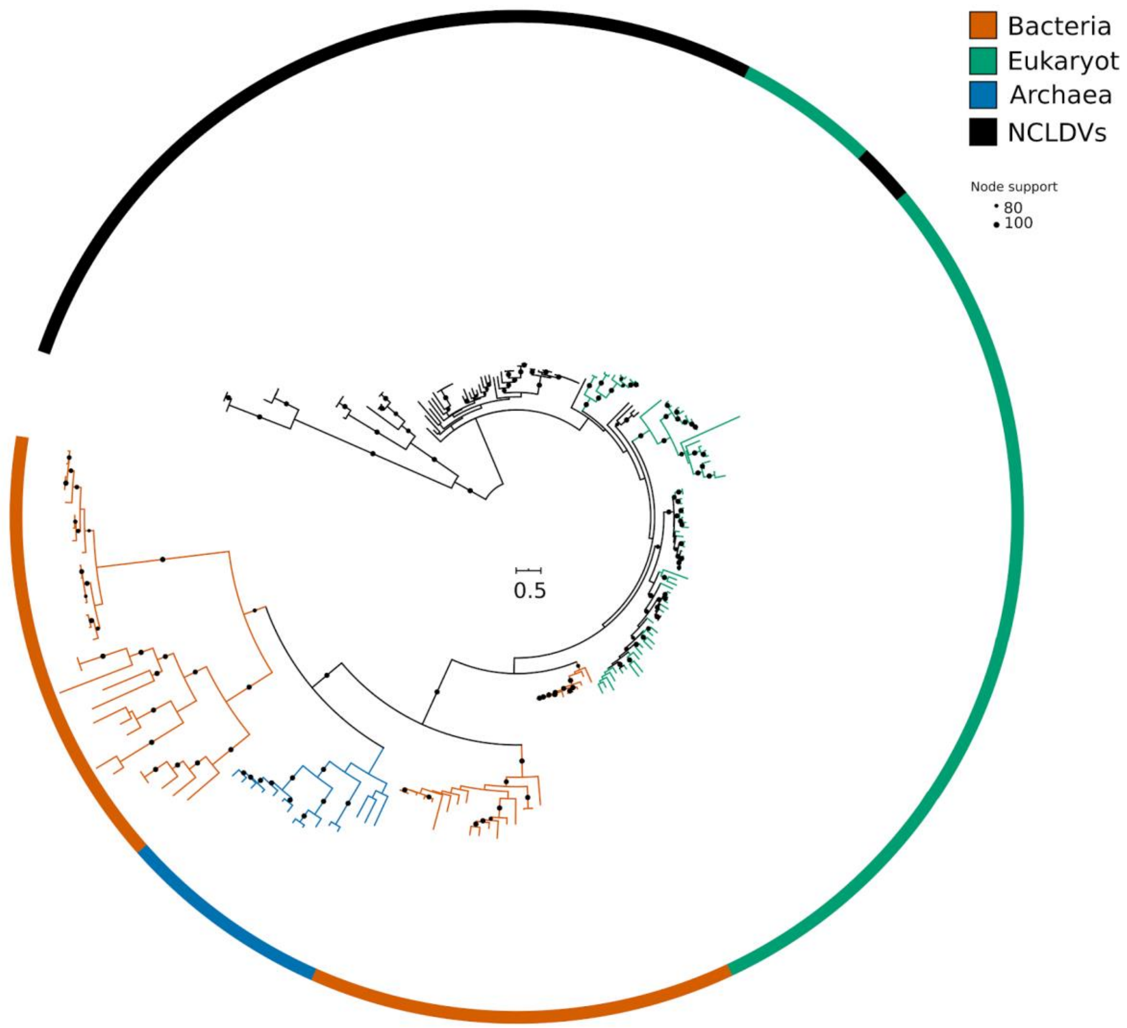

Supplementary Figure 11. Maximum-likelihood phylogenetic reconstruction of the TCA cycle enzyme Succinate dehydrogenase subunit B. Ultrafast bootstrap support >80% are shown.

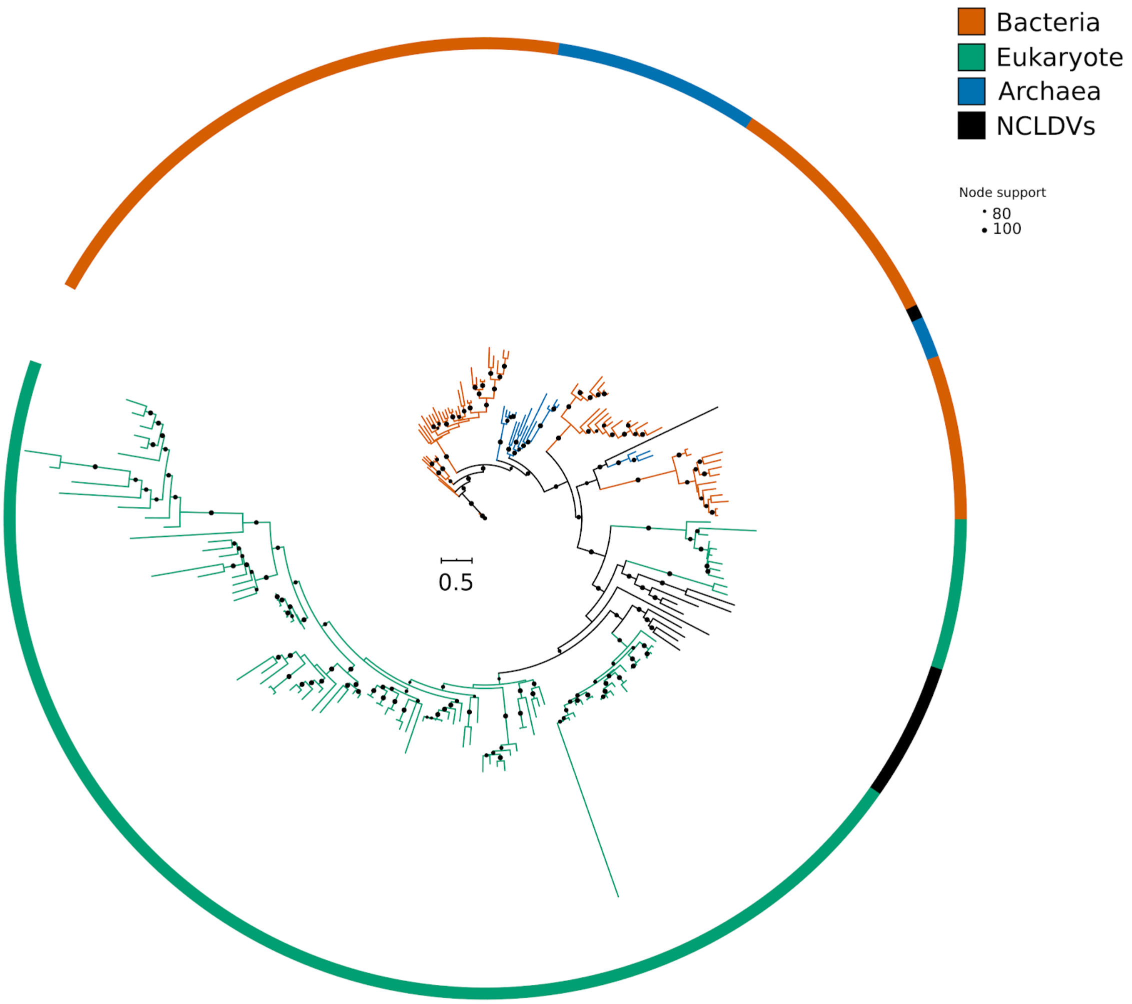

Supplementary Figure 12. Maximum-likelihood phylogenetic reconstruction of the iron storage and homoeostasis protein ferritin. Ultrafast bootstrap support >80% are shown.

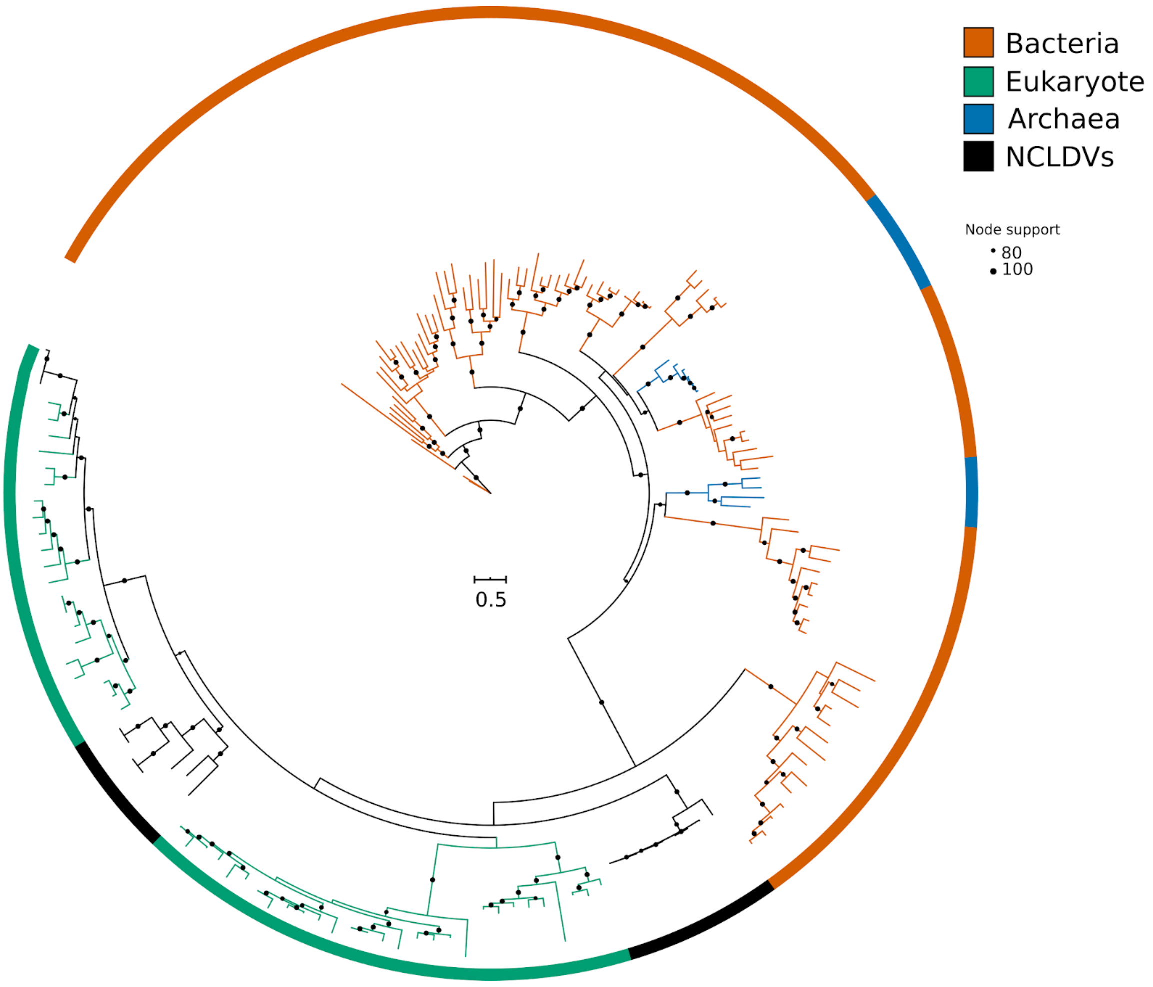

Supplementary Figure 13. Maximum-likelihood phylogenetic reconstruction of the Phosphate/Na+ symporter family proteins. Ultrafast bootstrap support >80% are shown.

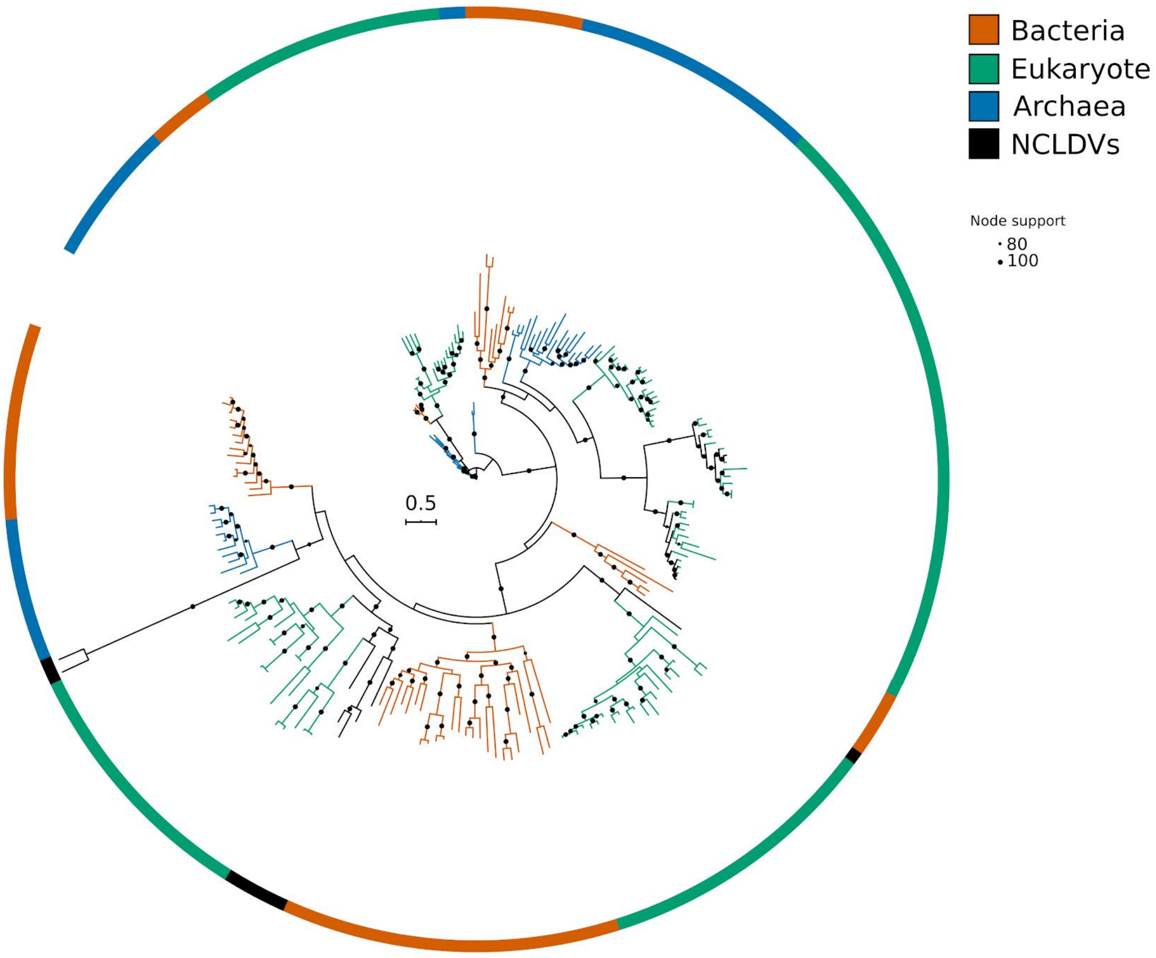

Supplementary Figure 14. Maximum-likelihood phylogenetic reconstruction of the Ammonium transporter (AmT) proteins. Ultrafast bootstrap support >80% are shown.

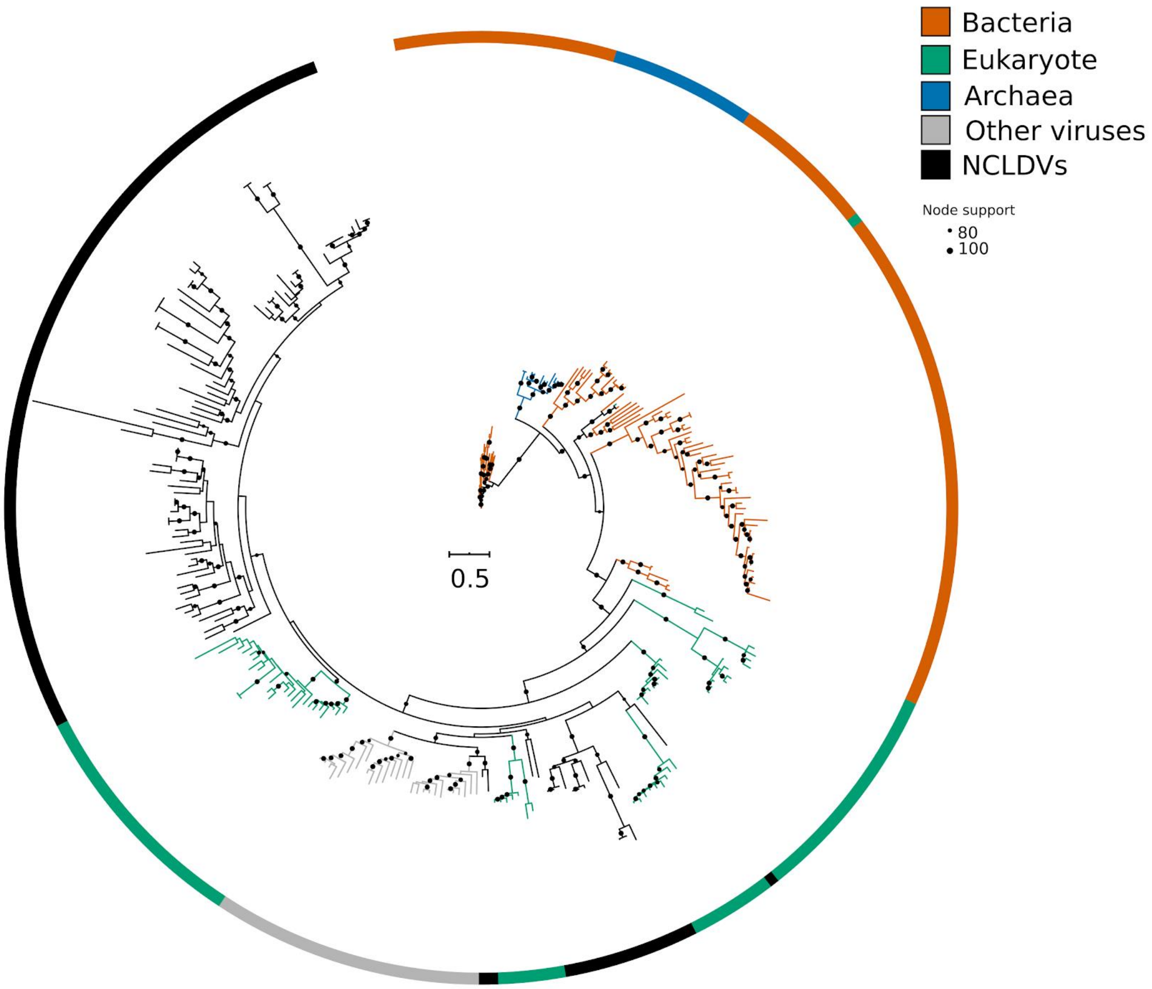

Supplementary Figure 15. Maximum-likelihood phylogenetic reconstruction of the antioxidant enzyme superoxide dismutase (SOD). Ultrafast bootstrap support >80% are shown. “Other viruses” refer to sequences from eukaryotic viruses other than NCLDVs.
