# Supplementary material for: Dynamic genome evolution and complex virocell metabolism of globally-distributed giant viruses
Source: Nat Commun. 2020 Apr 6;11:1710. doi: 10.1038/s41467-020-15507-2 (PMC7136201; doi:10.1038/s41467-020-15507-2)
Supplement: Supplementary file 3 — Description of Additional Supplementary Files [file 41467_2020_15507_MOESM3_ESM.docx]

**Supplementary Material.** This file contains supplementary figures for the manuscript.

**Supplementary Dataset 1.** This spreadsheet contains summary information for each NCLDV MAG reported in this study, including the genome size, number of encoded proteins, metagenome of origin, and other data. Additionally, this spreadsheet contains contig-level summary statistics for all decontamination analyses used in this study, and a list of all contigs that were removed due to similarity to cellular or bacteriophage sources. Lastly, this sheet contains a list of all reference NCLDV genomes used in this study.

**Supplementary Dataset 2.** This spreadsheet contains the annotation information for the orthologous groups calculated in this study. Only orthologous groups that exhibited similarity to a known protein family are listed here. Also provided are the enriched COG categories identified in each NCLDV clade.
